# In vitro and in vivo exploration of the cellobiose and cellodextrin phosphorylases panel in *Ruminiclostridium cellulolyticum*: implication for cellulose catabolism

**DOI:** 10.1186/s13068-019-1549-x

**Published:** 2019-09-03

**Authors:** Nian Liu, Aurélie Fosses, Clara Kampik, Goetz Parsiegla, Yann Denis, Nicolas Vita, Henri-Pierre Fierobe, Stéphanie Perret

**Affiliations:** 1 Aix-Marseille Univ, CNRS, LCB UMR 7283, 31 Chemin Joseph Aiguier, 13402 Marseille Cedex 20, France; 2 Aix-Marseille Univ, CNRS, BIP UMR 7281, Marseille, France; 3 Aix-Marseille Univ, CNRS, Plateforme Transcriptome, Marseille, France

**Keywords:** Cellobiose, Cellodextrins, Phosphorylase, Cellulolysis, Cellulose

## Abstract

**Background:**

In anaerobic cellulolytic micro-organisms, cellulolysis results in the action of several cellulases gathered in extracellular multi-enzyme complexes called cellulosomes. Their action releases cellobiose and longer cellodextrins which are imported and further degraded in the cytosol to fuel the cells. In *Ruminiclostridium cellulolyticum*, an anaerobic and cellulolytic mesophilic bacteria, three cellodextrin phosphorylases named CdpA, CdpB, and CdpC, were identified in addition to the cellobiose phosphorylase (CbpA) previously characterized. The present study aimed at characterizing them, exploring their implication during growth on cellulose to better understand the life-style of cellulolytic bacteria on such substrate.

**Results:**

The three cellodextrin phosphorylases from *R. cellulolyticum* displayed marked different enzymatic characteristics. They are specific for cellodextrins of different lengths and present different *k*_cat_ values. CdpC is the most active enzyme before CdpA, and CdpB is weakly active. Modeling studies revealed that a mutation of a conserved histidine residue in the phosphate ion-binding pocket in CdpB and CdpC might explain their activity-level differences. The genes encoding these enzymes are scattered over the chromosome of *R. cellulolyticum* and only the expression of the gene encoding the cellobiose phosphorylase and the gene *cdpA* is induced during cellulose growth. Characterization of four independent mutants constructed in *R. cellulolyticum* for each of the cellobiose and cellodextrin phosphorylases encoding genes indicated that only the cellobiose phosphorylase is essential for growth on cellulose.

**Conclusions:**

Unexpectedly, the cellobiose phosphorylase but not the cellodextrin phosphorylases is essential for the growth of the model bacterium on cellulose. This suggests that the bacterium adopts a “short” dextrin strategy to grow on cellulose, even though the use of long cellodextrins might be more energy-saving. Our results suggest marked differences in the cellulose catabolism developed among cellulolytic bacteria, which is a result that might impact the design of future engineered strains for biomass-to-biofuel conversion.

## Background

Cellulose is the most abundant polysaccharide produced on Earth and is constituted of linear chains of β-1,4-linked glucose units. It represents a large reservoir of glucose and an attractive renewable energy source. Nevertheless, glucose molecules are scarcely available from cellulose because of the tight crystalline packing of the cellulosic chains which makes this material recalcitrant to enzymatic degradation. Its biological deconstruction is, therefore, a limiting step in the carbon cycle on Earth and also a bottleneck in the process of biofuel or bio-based chemicals production [1].

Nonetheless, several anaerobic bacteria are able to use this recalcitrant substrate as the sole carbon and energy source [2]. Among them, *Ruminiclostridium cellulolyticum,* a mesophilic, anaerobic model bacterium raises special interest for years due to its ability to efficiently degrade and use plant cell wall polysaccharides including cellulose and hemicellulose, and the availability of genetic tools [3–8]. To achieve the enzymatic degradation of plant cell wall polysaccharides, it produces multi-enzyme complexes called cellulosomes by assembling on a scaffolding protein diverse enzymes belonging to families of glycoside hydrolase (GH), carbohydrate esterase (CE), or polysaccharide lyase (PL) [6, 9]. The released mono- and oligosaccharides are subsequently imported by the bacteria and catabolized. For example, the uptake of xyloglucan and cellodextrins was shown to be ensured by specific ABC transporters, the imported dextrins being further degraded into simple monosaccharides by cytosolic GHs. Two distinct clusters of genes dedicated to either the catabolism of xyloglucan or cellodextrins were shown to encode ABC transporter components (including a solute-binding protein collecting the solute to be imported and two transmembrane domains forming a channel), intracellular GH(s), and a signal transduction system [8, 10]. The ABC transporter called CuaABC (for cellulose utilization associated) has a solute-binding protein which binds to cellodextrins with lengths ranging from cellobiose (G2) to cellopentaose (G5), suggesting that at least these cellodextrins might be imported in the cytosol. CuaABC was shown to be essential for growth of the bacterium on both cellobiose and cellulose growth substrate [10]. We have formerly shown that imported cellobiose is subsequently converted into glucose and α-glucose 1-phosphate (G-1P) by the cellobiose phosphorylase A (CbpA) [10]. The gene encoding this enzyme forms with the genes *cuaABC* an operon named (*cuaABC*-*cbpA*). This operon is regulated by a predicted three-component system CuaDSR encompassing a binding protein CuaD, a sensor (CuaS), and a regulator (CuaR) encoded by the operon *cuaDSR* located upstream of *cuaABC*-*cbpA.* In our previous study, we constructed an *R. cellulolyticum cuaD* mutant strain (MTL*cuaD*) in which a type II intron inactivates *cuaD*. The modification induced a polar effect on the expression of the downstream genes *cuaS* (sensor) and *cuaR* (regulator), thus preventing the upregulation of the expression of *cuaABC*-*cbpA* operon encoding the cellodextrins ABC transporter and the cellobiose phosphorylase A. In consequence, the MTL*cuaD* strain was unable to grow on either cellobiose or cellulose. The transformation of the strain with a vector containing the ABC transporter genes but not the cellobiose phosphorylase-encoding gene *cbpA* restored growth on cellulose but not on cellobiose. This observation suggests that cellodextrins of degree of polymerization (DP) greater than 2 might be imported in the cytosol, thus ensuring growth on cellulose of this strain. Similarly, another cellulolytic strain (*Hungatei*) *Clostridium thermocellum* was reported to assimilate long cellodextrins of 5 and 6 glucose residues when grown on cellulose [11]. In general, the import of long cellodextrins is believed to be more cost-effective compared to the import of short ones, since for the same ATP transport cost, long cellodextrins carry more glucose units and, therefore, generate more energy than short ones [11].

In anaerobic cellulolytic bacteria, the cytosolic degradation of cellodextrins is usually ensured by cellobiose/cellodextrin phosphorylases [12, 13]. The cellodextrin phosphorylases catalyze reversible phosphorolysis reaction in which a β-1,4-glycosidic bond of a cellodextrin of n glucose units (called Gn with *n* ≥ 2) is cleaved in the presence of inorganic phosphate, releasing one G-1P from the non-reducing end and one G_n-1_ molecule. The phosphorylated glucose can directly enter the glycolysis pathway after conversion into glucose 6-phosphate (G-6P), without consumption of an ATP molecule for its phosphorylation, in contrast to the unphosphorylated glucoses generated by hydrolysis of cellodextrins. This pathway, therefore, represents an energetically more advantageous way of degrading oligosaccharides compared to hydrolysis, which is especially beneficial for anaerobic organisms [12–14]. The cellobiose phosphorylase A from *R. cellulolyticum* belongs to the GH94 Family. It is specific for cellobiose as well as other cellobiose phosphorylases described so far, with the exception of the cellobiose phosphorylase from *Thermosipho africanus* which is active on both cellobiose and long cellodextrins [10, 15–19]. If *R. cellulolyticum* is able to import long cellodextrins, enzyme(s) other than the cellobiose phosphorylase A might be implicated in their degradation. We analyzed the genome of *R. cellulolyticum* and identified three genes encoding putative cytosolic cellodextrin phosphorylases belonging to the GH94 family. We characterized the three cellodextrin phosphorylases and addressed the question of their role in the cellulose catabolism achieved by *R. cellulolyticum*.

## Results

### Characterization of the new cellodextrin phosphorylases

**Fig. 1:**
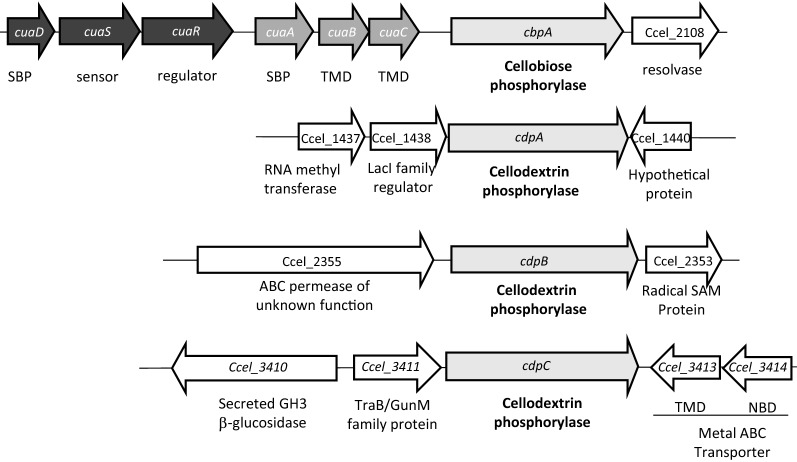
Cellobiose and cellodextrin phosphorylase genes and their neighboring genes. The genes encoding the putative cellobiose/dextrins phosphorylase are represented in light grey and surrounding genes are shown in white. The putative function of the gene products is indicated below, with SBP for Solute-Binding Protein, TMD for transmembrane domain of ABC transporter. The *cua* cluster is represented in black (signaling) and dark grey (ABC transporter)

The gene at the locus Ccel_2109 encodes the previously characterized cellobiose phosphorylase A [10]. It is located in the *cua* cluster, downstream of the genes *cuaABC* encoding an ABC transporter dedicated to the uptake of cellodextrins, and the genes *cuaDSR* encoding a putative three-component system involved in the signal transduction process. The genes at loci Ccel_1439, Ccel_2354, and Ccel_3412 encode three other phosphorylases belonging to the GH94 family, which are hereafter named CdpA, CdpB, and CdpC, respectively (Fig. 1). The three proteins lack a leader peptide and are, therefore, predicted to be cytosolic enzymes. The three genes are surrounded by genes not predicted to be related to cellulose degradation, or cellodextrin transport. Interestingly, the gene at the locus Ccel_1439 (*cdpA*) is located downstream of a gene encoding a regulator of the LacI family, suggesting that the latter protein could be involved in its regulation.

**Fig. 2:**
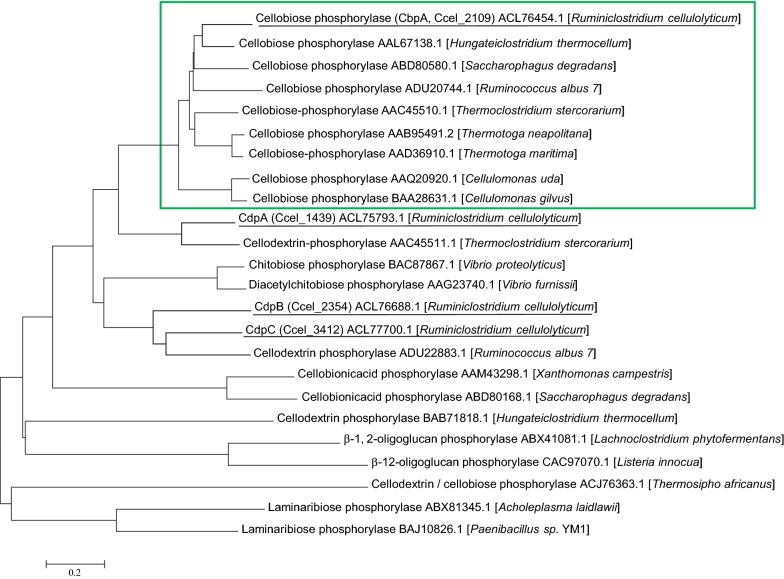
Phylogenetic analysis of the characterized GH94 phosphorylases. The phylogenetic tree was generated using neighbor joining analyses based on MEGA7 software (https://www.megasoftware.net/) with the characterized GH94 phosphorylases sequences. Branch lengths correspond to the evolutionary distances and represent the number of amino acid substitutions per site. Green box corresponds to the cellobiose phosphorylase cluster, and underscored proteins are the cellobiose and cellodextrin phosphorylases from *Ruminiclostridium cellulolyticum*

Phosphorylases which belong to the GH94 family include different enzymes specificities like cellobiose, cellodextrins, chitobiose, laminaribiose, and cellobionic acid (CAZy database, http://www.cazy.org/). A phylogenetic tree was generated based on the amino acid sequence from characterized bacterial GH94 phosphorylases. It encompasses phosphorylases active towards cellobiose, cellodextrins, chitobiose, laminaribiose, and cellobionic acid (Fig. 2). The phylogenetic analysis showed that the cellobiose phosphorylases together form a phylogenetic cluster. The cellodextrin phosphorylases, on the other hand, are—as formerly reported—located at a larger distance and do not form a cluster [19, 20]. CdpA is close to CepB from (*Thermo*) *Clostridium stercorarium,* whereas both CdpB and CdpC stand close to the cellodextrin phosphorylase RaCDP from *Ruminococcus albus* and form a new cluster distant from CdpA. Indeed, CdpA shares 70% identity with the cellodextrin phosphorylase CepB from *C. stercorarium*. On the other hand, CdpB and CdpC share 47% and 53% identity with the cellodextrin phosphorylase RaCDP from *Ruminococcus albus* [21], respectively.

To study their activities, recombinant enzymes containing a 6-His tag at their C-terminus were produced in the cytosol of *E. coli* and purified. The molecular size of the proteins analyzed by SDS-PAGE is in agreement with their theoretical molecular weight of 90.5, 91, and 94 kDa, respectively (Additional file 1). Activities were tested on cellodextrins varying from 2 to 5 glucose units and the catalytic parameters (*K*_m_ and *k*_cat_) for each enzyme were determined for their preferred substrate(s) (Table 1). None of these enzymes was active on cellobiose. CdpA was the most active on G4 and G5 with the highest activity measured on G5 and a residual activity detected on G3. CdpB preferably cleaved G4 cellodextrin but with a rather low activity compared to CdpA, whereas CdpC was highly active on G3 but poorly active on G4 and G5. Overall, the *K*_m_ values of the enzymes toward their preferred substrates were in the same range from 4.5 to 12 mM, which is higher than the *K*_m_ value of the cellobiose phosphorylase A for cellobiose (2.8 mM) [10]. In addition, these enzymes also exhibit significantly higher *K*_m_ values than those determined for other cellodextrin phosphorylases like *C. stercorarium* CepB which has *K*_m_ values ranging from 0.04 to 0.17 mM towards G3 to G5 [16], or *C. thermocellum* CtCDP which was reported to have *K*_m_ values around 0.8 mM for G3 and G4 (Table 2) [22]. Only the cellodextrin phosphorylase from *R. albus* (RaCDP) also exhibits quite high *K*_m_ values, in a similar range (2–6 mM). In *R. cellulolyticum*, CdpA and CdpC are the most active enzymes on G5 and G3, respectively, and are characterized by rather high *k*_cat_ and *K*_m_/*k*_cat_ values, especially CdpC. A similar pattern of activity was also described for RaCDP, which is the phylogenetically closest phosphorylase to CdpC. It displays similar *K*_m_ values and high *k*_cat_ (4500 to 5000 min^−1^) values (Table 2) [21]. Interestingly, cellodextrin phosphorylases from *R. cellulolyticum* preferentially degrade cellodextrins of specific lengths. The favorite substrate of CdpA is G5 followed by G4, whereas CdpB prefers G4, and CdpC shows a marked preference for G3. Such narrow specificities have not been described for any other cellodextrin phosphorylases characterized to date (Table 2). As the most active enzymes CdpA and CdpC have different cellodextrin length preferences, their coordinated action together with the cellobiose phosphorylase A should lead to the conversion of long dextrins like G5 into one glucose and four G-1P by sequential phosphorolysis.

**Table 1:** Catalytic parameters of the cellodextrin phosphorylases

	CdpA			CdpB			CdpC		
	*K*_m_	*k*_cat_	*k*_cat_/*K*_m_	*K*_m_	*k*_cat_	*k*_cat_/*K*_m_	*K*_m_	*k*_cat_	*k*_cat_/*K*_m_
G2	–	–	–	–	–	–	–	–	–
G3	Detected activity^a^: 15.2			Detected activity^a^: ≈ 0.4			5.4 ± 0.5	7650.8 ± 828.5	1410.4 ± 24.5
G4	11.9 ± 0.7	2340.1 ± 171.0	197.2 ± 7.3	4.7 ± 0.3	59.9 ± 5.4	12.8 ± 0.8	Detected activity^a^: ≈ 2		
G5	5.6 ± 0.6	2866.3 ± 140.0	515.2 ± 33.0	Detected activity^a^: ≈ 2			Detected activity^a^: ≈ 2		

*K*_m_ values are given in mM, *k*_cat_ values are given in min^−1^, and *k*_cat_/*K*_m_ values are given in min^−1^ mM^−1^

^a^In these experiments, *k*_cat_ or *K*_m_ value could not be determined due to low activity of the enzyme. The detected activities are given in µM min^−1^ and were measured using substrate at 1 mM, enzyme concentration at 1 µM, and incubation at 37 °C up to 24 h as in the case of CdpB and G3. (−) means no activity was detected. The data show the means and standard deviations of three independent experiments

**Table 2:** Catalytic parameters previously reported for cellodextrin phosphorylases from *R. albus*, *C. thermocellum,* and *C. stercorarium*

	RaCDP			CtCDP			CepB		
	*K*_m_	*k*_cat_	*k*_cat_/*K*_m_	*K*_m_	*k*_cat_	*k*_cat_/*K*_m_	*K*_m_	*k*_cat_	*k*_cat_/*K*_m_
G3	6.04	4572	713	0.81	240	296	0.04	162	4050
G4	4.16	5568	1338	0.82	192	234	0.05	414	8280
G5	2.41	5028	2086	nd	nd	nd	0.17	396	2329

RaCDP, cellodextrin phosphorylase from *R. albus* [21]; CtCDP, cellodextrin phosphorylase from *C. thermocellum* [22]; CepB, cellodextrin phosphorylase from *C. stercorarium* [16]. *K*_m_ value are given in mM, *k*_cat_ were converted from s^−1^ to min^−1^, and *k*_cat_/*K*_m_ values were calculated in min^−1^ mM^−1^. nd, not determined

### Modeling of the phosphorylases

**Fig. 3:**
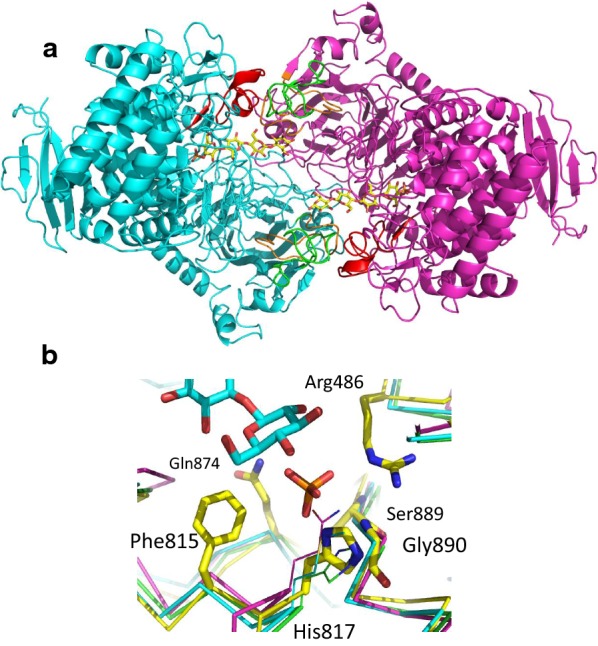
Structural modeling. **a** View of the homodimer model showing CdpC as an example. Cα cartoons of the monomers are colored in cyan and magenta, while loop regions L1, L2, and L3 are colored in red, green, and orange, respectively. The locations of the phosphate ions and the cellotetraose molecules as observed in 5NZ8 are indicated in yellow. **b** View of the phosphate-binding site. Side chains as observed in the structure of *C. thermocellum* cellodextrin phosphorylase (5NZ8) are indicated in sticks and are labeled. Cα ribbons of CdpA, CdpB, and CdpC are colored in green, cyan, and magenta, respectively. Replacement of His817 in 5NZ8 to His, Met, and Gln in CdpA, CdpB, and CdpC, respectively, are indicated in lines

Previous X-ray structure determination revealed that cellobiose and cellodextrin phosphorylases form homodimers in the asymmetric unit [23, 24]. To better understand observed differences in activity and substrate preference of the enzymes, we therefore built dimeric models of each of them using a three-step procedure as described in the Materials and Methods section (Fig. 3a). Even though side-chain orientations in the active site are only roughly similar in the generated models compared to the crystal structure of the substrate/cellodextrin phosphorylase complex of *C. thermocellum* (PDB code: 5nz8), these models permit to annotate the multiple sequence alignment of the four enzymes of *R. cellulolyticum* (Additional file 2) and they add a 3D perspective, useful in the search for clues on why they have differences in activity and substrate specificity. The structural conservation around the phosphorylation site is of special interest for the catalytic activity of the enzymes. In all four models, the phosphate-binding site is composed of three N-terminal regions of α-helices (α9, α18, and α21) and a C-terminal loop behind α-helix α8 and the N-terminal site of the center strand (β33) of a three-stranded antiparallel β-sheet. The sugar-binding site for the cellobiose at the non-reducing end of the cellodextrin substrate includes two additional loop regions. One of them is the prolongation of the N-terminus of helix α9, which is already involved in the phosphate site, and the other is the center part of a long loop connecting helices α13 and α14. All these structural elements involving the phosphorylation site are part of the same monomer. Their direct interaction sphere with either the phosphate ion or the phosphorylated sugar-binding site is mostly strictly conserved in all models (Table 3). An important exception is one residue of the phosphate ion coordination sphere which is a His residue in the cellobiose phosphorylase A and CdpA, but a Met in CdpB and a Gln in CdpC (Fig. 3b, Table 3).

**Table 3:** Conservation of active site residues

Enzyme	Phosphate site				Non-reducing end cellobiose site 1 and 2						
5NZ8	Arg 486	His 817	Gln 874	Ser 889	Arg 496	Glu 502	Asp624 NH	Cys 625	Trp 662 CO	Glu 810	Phe 815
CbpA	Arg 343	His 625	Gln 698	Thr 717	Arg 354	Asp 360	Asp 482 NH	Cys 484	Trp 480 CO	Glu 646	Phe 650
CdpA	Arg 331	His 628	Gln 674	Ser 693	Arg 342	Asp 348	Asp 474 NH	Thr 475	Trp 472 CO	Glu 621	Phe 626
CdpB	Arg 337	Met 637	Gln 683	Thr 702	Arg 347	Asp 354	Asp 486 NH	Cys487	Trp 484 CO	Glu630	Phe 635
CdpC	Arg 365	Gln 665	Gln 711	Thr 730	Arg 375	Asp 382	Asp 514 NH	Cys 515	Trp 512 CO	Glu 658	Phe 663

**Fig. 4:**
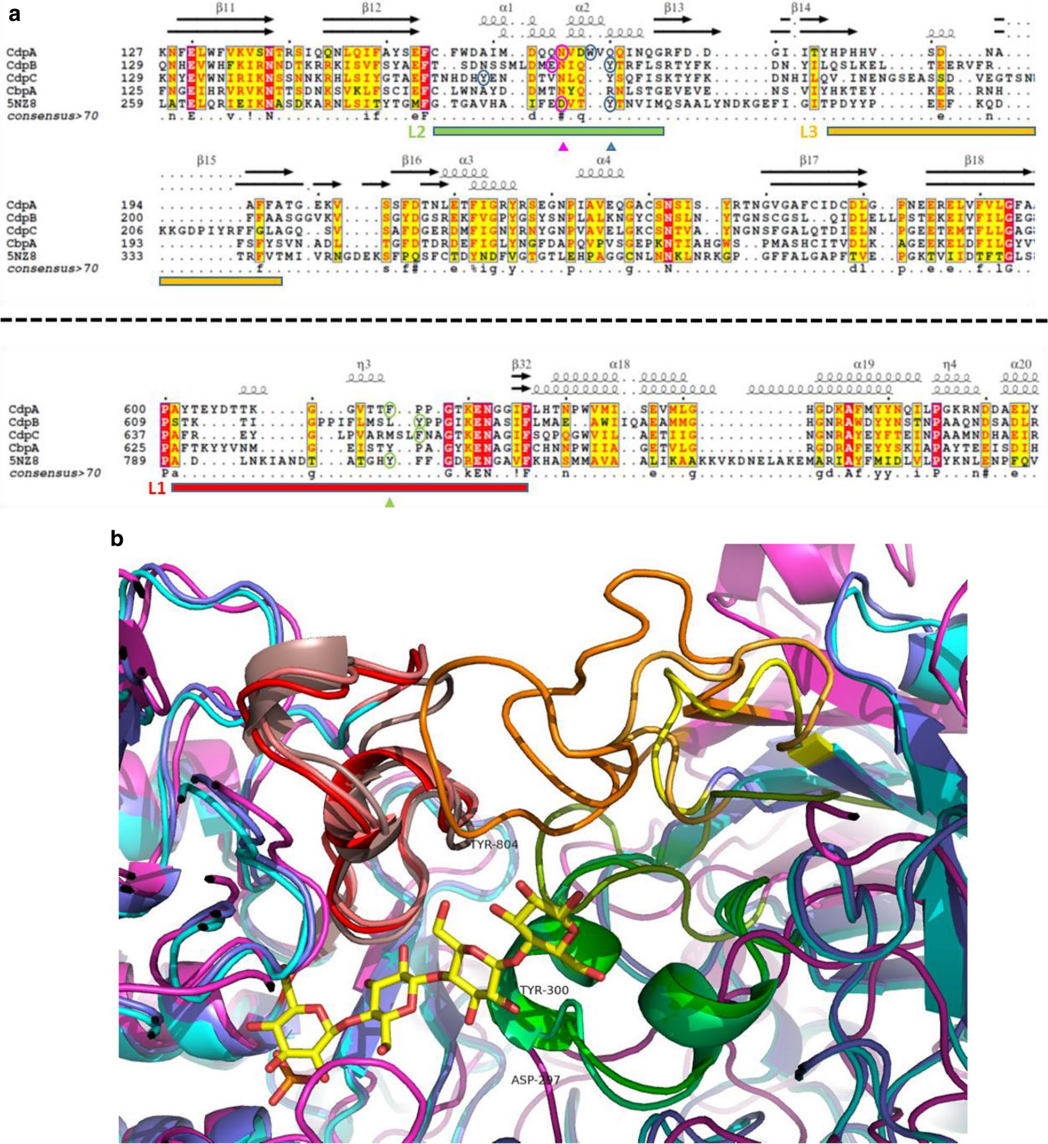
Modeling of the interface loops. **a** Extraction of parts of the sequence alignment of all modeled phosphorylases CdpA, CdpB, CdpC, and CbpA from *R. cellulolyticum* and *C. thermocellum* cellodextrin phosphorylase (5NZ8) as can be found in Additional file 2, showing the locations of the three loop regions L1, L2, and L3 which are forming the substrate-binding region beyond the cellobiose at the non-reducing end. Secondary structure from 5NZ8 (lower line) and the CdpA model (upper line) are also indicated, as well as locations of side chains Asp297 (magenta), Tyr300 (blue), and Tyr 804 (green) of 5NZ8 represented in triangles with their equivalent positions in the models as observed in the structural overlay represented in circles. **b** Zoom into the substrate-binding region of the Cα cartoon presentation of the modeled dimeric structures CdpA, CdpB, and CdpC in blue, cyan, and magenta, showing loop regions L1 in red, salmon, and light pink, L2 in green, blue–green, and olive, and L3 in yellow, light orange, and orange, respectively. The substrate in yellow is an overlay from 5NZ8

Substrate specificity for the sugar chain beyond cellobiose should be controlled by the presence of additional sugar-binding sites. In analogy to the cellodextrin phosphorylase/substrate complex of *C. thermocellum*, they are expected to extend into the interface of the homodimer. The region interacting with these sugar residues is constructed by three loop regions, which we will call L1, L2, and L3 (Fig. 4). L1 is located in the same monomer as the phosphate-binding site between helices α15 and α16, while loops L2 and L3 are located in the other monomer between β11 and β12 or β13 and β14. Variations in sequence composition and length of these loop regions allow the enzymes to create their distinct substrate-binding pocket. Sugar-binding sites are often stabilized by the presence of aromatic amino acids, what is also the case in *C. thermocellum* phosphorylase. The enzyme has several aromatic residues in the substrate pocket, namely Trp622, Phe815, Tyr804, and Tyr300 which is of particular interest as it forms a stacking interaction with the sugar chain between subsites 2 and 3. L1 and L2 have a similar length in all enzymes and contain a conserved aromatic residue. L1 contains Tyr804 (in *C. thermocellum*) which is also a Tyr in the cellobiose phosphorylase A and CdpA, or a Phe in CdpB and CdpC. L2 contains Tyr300 (in *C. thermocellum*) whose aromatic character is only conserved in all cellodextrin phosphorylase models (Trp in CdpA or Tyr in CdpB and CdpC) but not in the cellobiose phosphorylase A. The conservation of these aromatic residues can better be observed in the structural overlay of the models than in the sequence alignment, where they may be shifted even if their side chains are structurally close, as it is the case here. L2 also contains Asp297 which is another key residue for sugar binding and is involved in a salt bridge in the *C. thermocellum* enzyme. This salt bridge is not conserved in *R. cellulolyticum,* but the polar character of the site is maintained, since Asp297 is replaced by other polar residues. Finally loop L3 is short in CdpA and CdpB, but is 14–18 amino acids longer in CdpC. Even if low sequence identity with known 3D structure of these regions makes modeling difficult and the quality of their obtained 3D structure uncertain, it can be observed that the size of L1 and L2 is the same in all three Cdp-models allowing similar peptide backbone tracings. L3 is, however, significantly longer in CdpC which creates a more densely packed region after sugar subsite 3 in our model and might block the access of longer substrate chains. This could explain why CdpC is less active on substrates longer than three residues (Table 1). In contrast to the above observations on cellodextrin phosphorylase CdpC, blocking of substrates longer than cellobiose in cellobiose phosphorylase A is not accomplished by loops L1, L2, or L3, but by extending the loop between helices α13 and α14 (which is also involved in the stabilization of the cellobiose site as mentioned above).

### Biological role of the phosphorylases in *R. cellulolyticum*

**Fig. 5:**
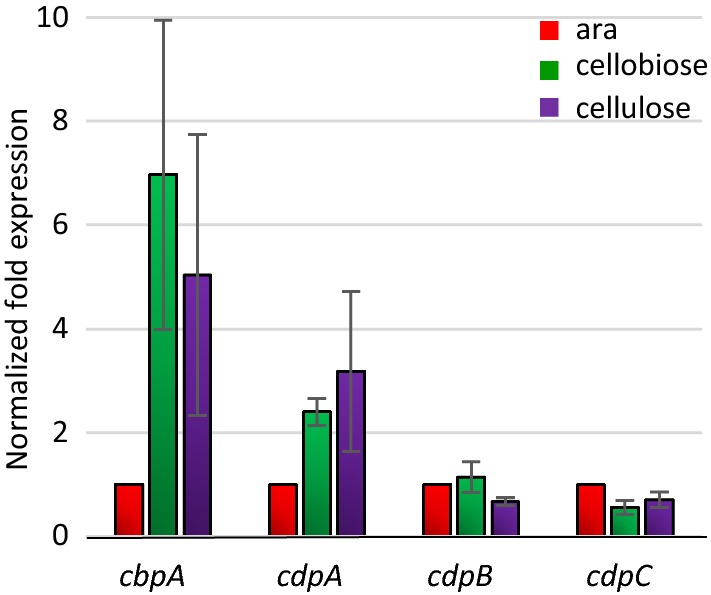
qPCR analysis of mRNA produced by WT strain. Total RNA was extracted from cultures of *R. cellulolyticum* grown in minimal medium supplemented with 0.2% arabinose, 0.2% cellobiose, or 0.5% cellulose as the sole carbon source. Normalization was performed using the 16S RNA-encoding gene. Error bars indicate the standard deviations of three independents experiments

To gain insights into the role of these enzymes in vivo, we first analyzed the expression levels of their corresponding gene in the wild-type (WT) *R. cellulolyticum* strain grown on arabinose, cellobiose, or cellulose as the carbon sources (Fig. 5). The expression level of each gene on arabinose served for data normalization. While *cdpB* and *cdpC* seem to be constitutively expressed in all three growth conditions, expression of the gene encoding the cellobiose phosphorylase (*cbpA*) and the gene *cdpA* is induced (from two to eight times) when the strain is grown in the presence of cellobiose or cellulose compared to arabinose, respectively.

**Fig. 6:**
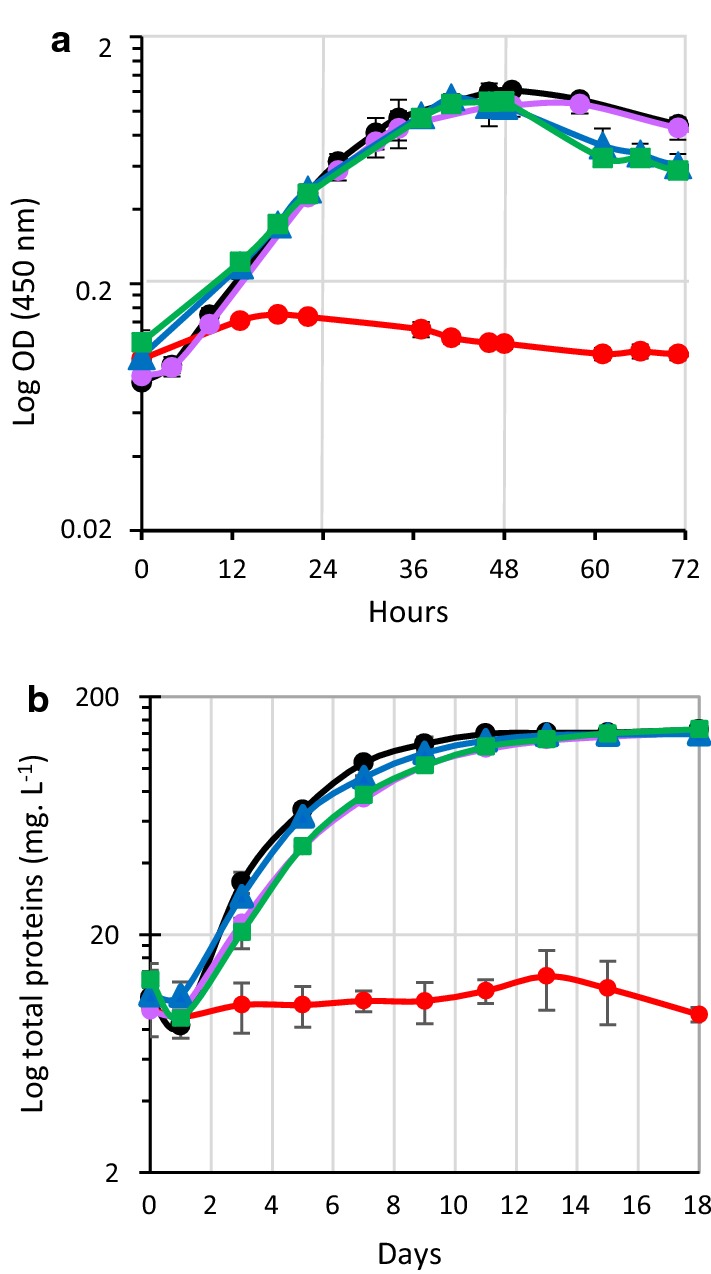
Growth of *R. cellulolyticum* wild-type and mutant *s*trains on cellobiose and cellulose. Wild-type (black) and the mutant strains MTL*cbpA* (red), MTL*cdpA* (purple), MTL*cdpB* (blue), and MTL*cdpC* (green) were grown in minimal medium-containing 2 g L^−1^ cellobiose (**a**) or 5 g L^−1^ cellulose (**b**). Experiments were performed in triplicate and bars indicate the standard deviations

The role of these enzymes in *R. cellulolyticum* was then addressed by the construction of four mutant strains, targeting the gene encoding the cellobiose phosphorylase (*cbpA*) and the genes *cdpA*, *cdpB,* and *cdpC*. We constructed the mutant strains using the Clostron insertional mutagenesis tool and obtained the strains MTL*cbpA*, MTL*cdpA*, MTL*cdpB,* and MTL*cdpC* [25]. Southern blot and PCR analyses showed a unique insertion at the expected location of the type II intron (Additional file 3). Growth of the four mutant strains on minimal medium was then tested on different carbon sources. When the medium was supplemented with arabinose, the growth of the mutant strains was comparable to that of WT strain (Additional file 4). With cellobiose as the carbon source, only strain MTL*cbpA* was unable to grow (Fig. 6a), which is consistent with previous results and confirms the essential role of the cellobiose phosphorylase A in cellobiose catabolism in *R. cellulolyticum* [10]. Inactivation of the other targeted genes encoding cellodextrin phosphorylases, on the other hand, did not impede or slow down growth on cellobiose, as could have been expected considering the activity pattern of these enzymes.

With cellulose as the carbon source, all the mutant strains, except MTL*cbpA,* were able to grow nearly as fast as the wild-type strain (Fig. 6b). In detail, growth of strain MTL*cdpB* was similar to the WT strain, suggesting that the gene *cdpB* plays only a minor role during the growth of *R. cellulolyticum*. This result is consistent with the enzymatic study of CdpB showing a very low activity and the expression study of *cdpB* gene, which is not induced on cellulose as the carbon source.

**Fig. 7:**
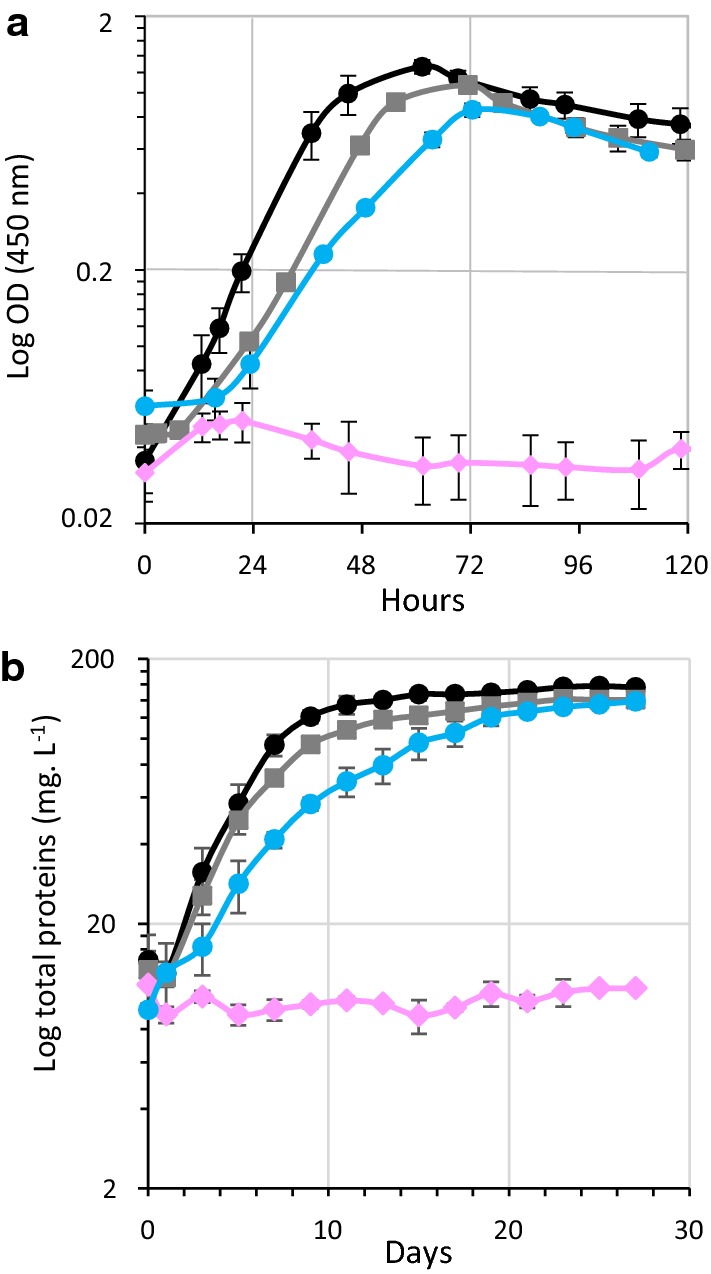
Growth of *R. cellulolyticum* wild-type and MTL*cbpA* derivative strains. Growth curve of different strains in minimal medium-containing either 2 g L^−1^ cellobiose (**a**) or 5 g L^−1^ cellulose (**b**). The strains are: WT strain (black), WT strain carrying an empty vector (grey), MTL*cbpA* strain carrying an empty vector (pink), and MTL*cbpA* strain carrying pSOS*cbpA* (blue). Experiments were performed in triplicate and bars indicate the standard deviations

Strains MTL*cdpA* and MTL*cdpC* grew slightly slower than the WT strain, though they reached the same final biomass, thereby indicating that genes *cdpA* and *cdpC* are more committed than *cdpB* in the degradation of cellodextrins. Nevertheless, their inactivation only had a minor impact on the fitness of the strain on cellulose. Importantly, this study emphasizes the central role of the cellobiose phosphorylase A in cellulose metabolism. A complementation study confirmed this observation: the transformation of the MTL*cbpA* mutant strain with a vector carrying the gene encoding the cellobiose phosphorylase A (pSOS956*cbpA*) indeed restored its growth on both cellobiose and cellulose. This was not the case when an empty control vector (pSOSzeroTm) was used for transformation (Fig. 7).

## Discussion

Cellulose degradation in cellulolytic bacteria is a complex process involving many different types of enzymes including cellulases, cellodextrins hydrolases, and phosphorylases. In the present work, we characterized and studied the role of three cellodextrin phosphorylases identified in *R. cellulolyticum* during its growth on cellulose.

The three enzymes are clearly different in terms of specificity and activity. Our modeling study revealed a difference at the phosphate ion coordination site, where a histidine conserved in the cellobiose phosphorylase A and CdpA is replaced by a Met in CdpB and a Gln in CdpC. The variations observed at this critical position might be important for the activity of the enzymes, because it changes the coordination of the phosphate ion. The weak activity observed for CdpB may in part be due to the replacement of the His by a Met in CdpB. In CdpC, which is the most active of the enzymes studied, the His is replaced with a Gln. Interestingly, the presence of a Gln in the same position was previously reported for the cellodextrin phosphorylase from *R. albus* (RaCDP), which is also the most similar enzyme to CdpC among all previously characterized phosphorylases [21]. The mutation of this Gln to a His in RaCDP increased its affinity for inorganic phosphate, but decreased the *k*_cat_ of the variant by ten times compared to the wild-type enzyme. To our knowledge, this enzyme and CdpC are the most active cellodextrin phosphorylases reported to date (Tables 1, 2). The presence of the Gln in the phosphate coordination seems to be a key amino acid for the high activity of these enzymes.

Cellodextrin phosphorylases already characterized to date display similar activity levels on cellodextrins of various lengths (Table 2). In contrast, CdpA, CdpB, and CdpC are more restrictive and specifically phosphorolyse cellodextrins of a particular degree of polymerization. Our modeling studies highlight the importance of the length of loop L3. Its long size in CdpC could lead to the constrained specificity of the enzyme for short dextrins like cellotriose. Its reduced size in CdpA might enable the enzyme to bind and process long cellodextrins like G4 and G5. Nevertheless, it remains unclear why G3 is such a poor substrate for CdpA and why CdpB is specific for G4.

In vivo studies were also performed to evaluate the role of the cellobiose or cellodextrin phosphorylases during growth on cellulose. As mentioned in the introduction, we previously found that the *R. cellulolyticum* MTL*cuaD* mutant transformed with a vector containing only *cuaABC* but not *cdpA* was able to grow on cellulose but not on cellobiose, suggesting that cellodextrins longer than G2 have sustained its growth even in the absence of the cellobiose phosphorylase A on cellulose [10]. However, direct inactivation of the gene *cbpA* showed that the cellobiose phosphorylase A was critical for growth on both cellobiose and cellulose, thus suggesting that the presence of longer cellodextrins does not contribute importantly to growth on cellulose. These contradictory results could be explained by a specific regulation of the gene *cbpA* encoding the cellobiose phosphorylase, which is not directly inactivated in the MTL*cuaD* (pSOS*cuaABC*) strain. The chromosomic expression of *cbpA* might, indeed, be induced specifically when this strain is grown on cellulose but not on cellobiose (as this strain cannot grow on the disaccharide). This regulation might depend on the presence of cellodextrins larger than G2 that are only present during growth on cellulose. The intergenic region between the genes *cuaC* and *cbpA* is 700 bp long, and might carry regulatory sequence(s) involved in the specific induction of the expression of *cbpA*. The specific regulation of the expression of the gene encoding the cellobiose phosphorylase in the presence of long cellodextrins might be necessary to ensure their complete degradation into glucose and glucose 1-*P*. The cleavage of G2 is indeed the final step in the cellodextrin degradation pathway, and our data show that the cellobiose phosphorylase is the only cytosolic enzyme performing cellobiose breakdown in *R. cellulolyticum*.

The inactivation of the gene encoding the cellobiose phosphorylase in *R. cellulolyticum* totally blocks its growth on cellulose, but the independent inactivation of each of the three cellodextrin phosphorylases has only a minor effect. This result might be explained by other cytosolic enzyme compensation. Indeed, in each single mutant, the genes encoding the two other cellodextrin phosphorylases remained intact, whose expression might, together with other intracellular glycoside hydrolases, contribute to the degradation of long cellodextrins. The genome indeed contains four additional predicted intracellular GH, one GH of family 1 (encoded at the locus Cel_0374), and 3 GH of family 3 (encoded at the respective locus Ccel_0203, Ccel_1139 and Ccel_2454). Among them, the GH3 (locus Ccel_2454) and the GH1 (locus Ccel_0374) were reported to be poorly active on cellodextrins, and the expression of the gene at the locus Ccel_1139 appears to be specifically induced when the strain is grown in xylan but not in cellulose or corn stover containing medium, suggesting that they are probably not related to the intracellular degradation of the cellodextrins [7, 8, 26]. Only the GH3 encoded at the locus Ccel_ 0203 could be involved. It is predicted to be a β-xylosidase and the expression of the corresponding gene at the locus Ccel_0203 is three-to-four times more induced in medium-containing cellulose, cellobiose, xylan, and corn stover compared to monosaccharide-based media [6, 7]. The product of this gene will need to be characterized in the future to clarify its possible role in cellulose catabolism in *R. cellulolyticum*.

The cellobiose phosphorylase A is essential for both cellobiose and cellulose catabolism in *R. cellulolyticum* and considering that this enzyme acts in the final common step in the degradation pathways of all longer cellodextrins, its importance seems consistent. However, the inactivation of *cbpA* produced a dramatic effect on growth on cellulose, even though the other phosphorylases and cytosolic GH genes were intact. Their action on G5, G4, or G3 in the cytosol should also have fueled the cell with G-1P and/or glucose to sustain its growth. A reasonable explanation for this unexpected strong impact of the cellobiose phosphorylase A on the growth of the bacterium on cellulose could be that long cellodextrins (> G2) are probably scarcely imported, whereas cellobiose might be the main imported sugar which sustains growth on cellulose. This hypothesis is supported by other data: (i) cellobiose was shown to be the most abundant sugar to be released by the action of cellulosomes on cellulose in vitro [6], (ii) an additional cell surface enzyme, Cel5I, is highly active on cellodextrins or cellulose, releasing mainly cellobiose from cellulosic substrates at the vicinity of the cell [27], (iii) CuaA, which is the binding protein of the main ABC importer for cellodextrins in *R. cellulolyticum*, binds with a greater affinity to short cellodextrins than to longer ones (G2 > G3 > G4 > G5), thus probably favoring the import of cellobiose which is, in addition, the major product released by cellulosomes as mentioned above [10], (iv) *K*_m_ values of the most active cellodextrin phosphorylase of *R. cellulolyticum* for cellodextrins are at least twice as high as the *K*_m_ value of the cellobiose phosphorylase A for cellobiose, in contrast to *C. thermocellum* whose cellodextrin phosphorylase has an apparent lower *K*_m_ (0.61 mM) for cellodextrins than the cellobiose phosphorylase (3.3 mM) for cellobiose [13] and which was shown to import rather long cellodextrins during growth on cellulose [11].

All these observations suggest that *R. cellulolyticum* favors the import and catabolism of cellobiose rather than longer cellodextrins when grown on cellulose. This difference in sugar uptake of *R. cellulolyticum* compared to *C. thermocellum* is difficult to explain considering that cellobiose uptake is less energetically advantageous than that of longer cellodextrins in terms of ATP consumed/imported molecule, what is especially important for strict anaerobic bacteria. The reason why *R. cellulolyticum* seems to adopt a “short” dextrin strategy although it is an anaerobic organism could be related to the localization of its cellulosomes with respect to the cells. Indeed, in the thermophile *C. thermocellum,* the major cellulosomal scaffolding protein is tethered to the cell surface, mediating the binding of the cells to the cellulose fibers. This narrow space between cells and cellulosomes might reduce the diffusion of the long cellodextrins directly released in the vicinity of the cell, and facilitate their direct assimilation [11]. In contrast, no evidence has ever been reported that cellulosomes produced by *R. cellulolyticum* are located at the bacterial cell surface [28]. As a consequence, cellulolysis performed by its cellulosomes might occur remotely in *R. cellulolyticum* compared to *C. thermocellum*. This larger cellulosomes-to-cell distance may prevent the cells from importing intermediate degradation products (like the long cellodextrins) and favor a more complete degradation into cellobiose as the final product, which is ultimately imported by *R. cellulolyticum*. Overall, our results suggest differences in the cellulose catabolism strategies developed by cellulolytic bacteria, for which the extracellular cellulose degradation and cellodextrins import and intracellular degradation steps are fine-tuned.

## Conclusion

In the present study, three cellodextrin phosphorylases produced in *R. cellulolyticum* were characterized. They display different specificities and activities towards cellodextrins of various length. Through the study of the corresponding mutant strains and derivatives strains, the cellobiose phosphorylase was shown to play an essential role during growth on cellobiose and on cellulose. The results suggest that cellobiose is the major dextrin which sustains growth in *R. cellulolyticum* and reveal for this strain an alternative strategy in anaerobic cellulose catabolism compared to *C. thermocellum*. Future designs of engineered strains performing biomass-to-biofuel conversion might benefit from these findings.

## Materials and methods

### Strains and media vectors

Strains and vectors used in this study are reported in Additional file 5. *Escherichia coli* strains were grown at 37 °C in Lysogenic–Broth medium supplemented with the appropriate antibiotic (100 µg mL^−1^ of ampicillin or 35 µg mL^−1^ of chloramphenicol). *R. cellulolyticum* H10 ATCC 35319 [29] was grown anaerobically at 32 °C in minimal medium [30] supplemented with either 2 g L^−1^ cellobiose, arabinose, or 5 g L^−1^ crystalline cellulose type 20 (Sigmacell, Sigma-Aldrich, Saint Louis, MO). Growth in cellobiose or arabinose-supplemented basal medium was followed by monitoring optical density at 450 nm over time. When cultured on 5 g L^−1^ crystalline cellulose, growth was monitored by measurement of the total protein content as described previously [6].

Primers used in this study are reported in Additional file 6.

### Quantitative real-time-PCR for transcriptional analyses

Cultures of *R. cellulolyticum* grown in minimal medium supplemented with arabinose (2 g L^−1^), cellobiose (2 g L^−1^) or cellulose (5 g L^−1^) were harvested at mid- or late-exponential phase of growth (8000 *g* 10 min). Total RNAs were isolated and cDNAs were synthesized as previously described [10]. qPCR analyses were performed on cDNA using primers listed in Additional file 6, as previously described [10]. qPCR was carried out on CFX96 real-time PCR detection system (Bio-Rad) and the result was analyzed using the Bio-Rad CFX manager software, v3.1 (Bio-Rad). The 16S RNA-encoding gene was used as a reference for normalization. For each point, a biological triplicate and a technical duplicate were performed. The amplification efficiencies for each primer pairs were comprised between 80 and 100%.

### Cloning of the genes encoding rCdpA, rCdpB, and rCdpC in *E. coli*

rCdpA, rCdpB, and rCdpC were designed to contain six histidine residues at their C-terminus. All genes were amplified by PCR using the genomic DNA of *R. cellulolyticum* as the matrix. For *cdpA*, the products of the PCR obtained using the primer pairs 1439NdeIdir/1439_a951t_rev and 1439_a951t_dir/1439XhoIrev were used as template to produce the final overlapping amplicon using the 1439NdeIdir/1439XhoIrev primers pairs. For the genes *cdpB* and *cdpC*, the primers pairs 2354NdeIdir/2354XhoIrev and 3412NdeIdir/3412XhoIrev were used to produce the corresponding amplicon, respectively. The three amplicons were subsequently digested with *Nde*I and *Xho*I and cloned into a *Nde*I–*Xho*I linearized pET22b(+), thereby generating the pET-*cdpA* pET-*cdpB* and pET-*cdpC*. The plasmids were verified by sequencing and used to transform the BL21 (DE3) strain to overproduce the corresponding recombinant proteins.

### Production and purification of the recombinant proteins

Recombinant *E. coli* BL21 (DE3) strains were grown at 37 °C with shaking to an optical density at 600 nm of 1.5, isopropyl-β-d-thiogalactopyranoside (IPTG) was added to a final concentration of 150 µM, and the cultures were incubated overnight under shaking at 18 °C. The cells were then harvested by centrifugation for 10 min at 3000*g* and the cell pellet was suspended in 30 mM Tris–HCl (pH 8) added with 5 mM imidazole and a few DNase I (Sigma-Aldrich, USA), and broken in a French press. After centrifugation of the crude extract (10 min, 4 °C, 10,000*g*), the supernatant containing his-tagged proteins was loaded onto a column of Ni-nitrilotriacetic acid resin (Thermofisher USA) equilibrated with 30 mM Tris–HCl (pH 8) 5 mM imidazole. Elution was performed using 30 mM Tris–HCl (pH 8) 100 mM imidazole. The eluted proteins were loaded on an ion-exchange chromatography column (Mono Q 4.6/100 PE, GE Healthcare, USA), equilibrated with 30 mM Tris–HCl (pH 8), and then eluted by a linear NaCl gradient (0–0.5 M). The purified proteins were dialyzed by ultrafiltration at 4 °C (Vivaspin 20, 30 kDa cutoff, Sartorius, Germany) with 25 mM potassium phosphate buffer (pH 7). The absorbance at 280 nm was measured and the protein concentration was determined using their specific extinction coefficient (CdpA, 168,290 M^−1^ cm^−1^; CdpB, 159,810 M^−1^ cm^−1^; CdpC, 166,800 M^−1^ cm^−1^) calculated from online program (https://web.expasy.org/protparam/).

### Phosphorylase activity measurement

For enzymatic parameter measurements, the enzymes were incubated with substrates (Megazyme) in 50 mM phosphate buffer (pH 7) containing 0.01% (w/v) NaN_3_ at 37 °C (for detailed information, see Additional file 7). Then, 200 µL of sample was mixed with 50 µL of 0.5 M sodium hydroxide were added prior to analyses by High-Pressure Anion Exchange Chromatography coupled with Pulsed Amperometric Detection (HPAEC-PAD). 25 µL were applied to a Dionex CarboPac PA1 column (4 × 250 mm) and the corresponding guard column (4 × 50 mm) at 30 °C. Sugars were eluted using solutions A (0.1 M NaOH) and B (0.5 M sodium acetate, 0.1 M NaOH). For glucose, α-d-glucose-1-phosphate, and cellodextrins quantifications, the following multi-step procedure was used: isocratic separation (5 min, 95% A + 5% B), separation gradient (8 min, 10 to 37% B), column wash (2 min, 99% B), and subsequent column equilibration (2.5 min, 95% A + 5% B). The flow rate was kept at 1 mL min^−1^ in all cases. Injection of samples containing glucose, α-d-glucose-1-phosphate, cellobiose, cellotriose, cellotetraose, and cellopentaose at known concentrations (ranging from 4 to 100 µM) was used to identify and quantify the released sugars. Calculation of *k*_cat_ and *K*_m_ is based on Lineweaver–Burk method.

### Mutant construction and complementation of MTL*cbpA* strain

Gene inactivation was performed using the ClosTron technology as previously described [25, 28]. We used the Perutka algorithm (http://ClosTron.com) to choose the integration sites in the target genes and to generate the primers sequence used to retarget the Ll.LtrB intron in the pMTL007 [IBS, EBS1d, and EBS2] (Additional file 6). The sets of primers aiming to independently inactivate the genes *cbpA*, *cdpA*, *cdpB,* and *cdpC* were used to produce an amplicon by overlapping PCR using pMTL007 as the matrix. The amplicons and the pMTL007 were both digested with *Bsr*GI and *Hin*dIII and ligated to generate the pMTL*cbpA*, pMTL*cdpA,* pMTL*cdpB,* and pMTL*cdpC* used for transformation of *R. cellulolyticum*. After in vitro methylation with MspI methylase, the vectors were transferred in *R. cellulolyticum* by electro-transformation as previously described [3, 4]. Thiamphenicol-resistant clones carrying replicative pMTL*cbpA*, pMTL*cdpA,* pMTL*cdpB,* or pMTL*cdpC* were selected. In a second step, the integration event was selected in erythromycin-containing basal medium. The resulting modified strains interrupted in either *cbpA*, *cdpA*, *cdpB,* or *cdpC* were called MTL*cbpA*, MTL*cdpA,* MTL*cdpB,* and MTL*cdpC*, respectively.

Southern blot was performed as described in Blouzard et al. [6]. Genomic DNAs purified from the MTL*cbpA*, MTL*cdpA,* MTL*cdpB,* and MTL*cdpC* mutant and wild-type *R. cellulolyticum* strain were digested with *Eco*RI or *Pst*I and hybridized with a labeled probe targeting erythromycin marker gene after migration. The insertion site of the intron was checked by PCR analysis performed using primers hybridizing upstream and downstream of the targeted site (Additional file 3).

For complementation studies, the vectors pSOSzeroTm and pSOS*cbpA*, already constructed in a previous study [10, 31] were transferred in the MTL*cbpA* mutant strain as previously described [10].

### Modeling studies

3D models of the homodimeric structures were generated using three steps. First, the I-TASSER modeling server was used for the construction of models of the monomers [32, 33]. Then, two monomers were assembled to homodimers by overlaying them to the homodimer complex of the *C. thermocellum* cellodextrin phosphorylase with cellotetraose (pdb code: 5nz8) [24] using the Wincoot software [34]. Finally, homodimers were refined using the FG-MD server to eliminate side-chain collisions and refine the interface [35]. The phosphate ion and the cellotetraose were inserted by overlay using their location in the cellodextrin phosphatase complex. Sequence alignment was performed using the T-coffee server [36] including sequence and structural data from the *C. thermocellum* cellodextrin phosphatase structure (pdb code: 5nz8). The alignment was processed for publication using the ESPRIPT server v 3.0 [37].

## Supplementary information

**Additional file 1.** Purified recombinant phosphorylases. Samples of purified recombinant proteins (3 µg) were loaded on gradient 4–15% SDS-PAGE then Coomassie Blue stained. The recombinant cellobiose phosphorylase (CbpA) and CdpA, CdpB and CdpC have theoretical molecular weights of 93.5 kDa, 90.5, 91 and 94 kDa, respectively.  

**Additional file 2.** Sequence alignment of all modeled phosphorylases CdpA, CdpB, CdpC and CbpA from *R. cellulolyticum* and *C. thermocellum* cellodextrin phosphorylase (5NZ8). Secondary structure from 5NZ8 (lower line) and the CdpA model (upper line) are also indicated.

**Additional file 3.** Molecular analysis of the *Ruminiclostridium cellulolyticum* mutant strains. A. Southern blot analysis of the strains. Genomic DNA or pMTL*cbpA* and pMTL*cdpB* were digested by *Pst*I or *Eco*RI. After migration and transfert, the membrane was probed with a labeled probe targeting the erythromycine resistance cassette. The size of the detected fragments is consistent with theoretical sizes: MLT*cbpA*, 2.8 kb; MTL*cdpA*, 6.6 kb; MTL*cdpB*, 4.2 kb; MTL*cdpC*, 7.8 kb. B. PCR analysis of genomic DNA using primers hybridizing upstream and downstream the insertion site in the respectives target genes. Insertion of the intron increases the size by 1,78 kb in the mutant strains compared to the WT genomic DNA.

**Additional file 4.** Growth of *R. cellulolyticum* wild-type, mutant and derivatives strains on arabinose The strains were grown on minimal medium containing 2 g L^−1^ arabinose. A. the strains are: WT (black) and mutant strains MTL*cbpA* (red), MTL*cdpA* (purple), MTL*cdpB* (blue) and MTL*cdpC* (green). B. The strains are: WT strain (black), WT strain carrying an empty vector (grey), MTL*cbpA* strain carrying an empty vector (pink), MTL*cbpA* strain carrying pSOS*cbpA* (blue). Experiments were performed in triplicates and bars indicate standard deviation.

**Additional file 5.** Bacterial strains and vectors used.

**Additional file 6.** Primer sequences used in the present study.

**Additional file 7.** Experimental conditions used for enzymatic parameter measurement. The tables show the initial velocities measured for each cellodextrin phosphorylase and substrate using experimental conditions obtained after optimization of the enzyme concentrations and the time points.

## Data Availability

The data sets used and/or analyzed during the current study are available from the corresponding author on reasonable request.

## References

[1] Lynd LR, Weimer PJ, van Zyl WH, Pretorius IS (2002). Microbial cellulose utilization: fundamentals and biotechnology. Microbiol Mol Biol Rev.

[2] Koeck DE, Pechtl A, Zverlov VV, Schwarz WH (2014). Genomics of cellulolytic bacteria. Curr Opin Biotechnol.

[3] Jennert KC, Tardif C, Young DI, Young M (2000). Gene transfer to *Clostridium cellulolyticum* ATCC 35319. Microbiology.

[4] Tardif C, Maamar H, Balfin M, Belaich JP (2001). Electrotransformation studies in *Clostridium cellulolyticum*. J Ind Microbiol Biotechnol.

[5] Perret S, Bélaich A, Fierobe HP, Bélaich JP, Tardif C (2004). Towards designer cellulosomes in Clostridia: mannanase enrichment of the cellulosomes produced by *Clostridium cellulolyticum*. J Bacteriol..

[6] Blouzard JC, Coutinho PM, Fierobe HP, Henrissat B, Lignon S, Tardif C, Pages S, de Philip P (2010). Modulation of cellulosome composition in *Clostridium cellulolyticum*: adaptation to the polysaccharide environment revealed by proteomic and carbohydrate-active enzyme analyses. Proteomics.

[7] Xu C, Huang R, Teng L, Wang D, Hemme CL, Borovok I (2013). Structure and regulation of the cellulose degradome in *Clostridium cellulolyticum*. Biotechnol Biofuels.

[8] Ravachol J, de Philip P, Borne R, Mansuelle P, Maté MJ, Perret S (2016). Mechanisms involved in xyloglucan catabolism by the cellulosome-producing bacterium *Ruminiclostridium cellulolyticum*. Sci Rep..

[9] Cantarel BL, Coutinho PM, Rancurel C, Bernard T, Lombard V, Henrissat B (2009). The Carbohydrate-Active EnZymes database (CAZy): an expert resource for Glycogenomics. Nucleic Acids Res.

[10] Fosses A, Maté M, Franche N, Liu N, Denis Y, Borne R (2017). A seven-gene cluster in *Ruminiclostridium cellulolyticum* is essential for signalization, uptake and catabolism of the degradation products of cellulose hydrolysis. Biotechnol Biofuels.

[11] Zhang YH, Lynd LR (2005). Cellulose utilization by *Clostridium thermocellum*: bioenergetics and hydrolysis product assimilation. Proc Natl Acad Sci USA..

[12] Lou J, Dawson KA, Strobel HJ (1997). Cellobiose and cellodextrin metabolism by the ruminal bacterium *Ruminococcus albus*. Curr Microbiol.

[13] Zhang YH, Lynd LR (2004). Kinetics and relative importance of phosphorolytic and hydrolytic cleavage of cellodextrins and cellobiose in cell extracts of *Clostridium thermocellum*. Appl Environ Microbiol.

[14] Ha SJ, Galazka JM, Joong OhE, Kordić V, Kim H, Jin YS (2013). Energetic benefits and rapid cellobiose fermentation by *Saccharomyces cerevisiae* expressing cellobiose phosphorylase and mutant cellodextrin transporters. Metab Eng.

[15] Sasaki T, Tanaka T, Nakagawa S, Kainuma K (1983). Purification and properties of *Cellvibrio gilvus* cellobiose phosphorylase. Biochem J..

[16] Reichenbecher M, Lottspeich F, Bronnenmeier K (1997). Purification and properties of a cellobiose phosphorylase (CepA) and a cellodextrin phosphorylase (CepB) from the cellulolytic thermophile *Clostridium stercorarium*. Eur J Biochem.

[17] Hamura K, Saburi W, Abe S, Morimoto N, Taguchi H, Mori H, Matsui H (2012). Enzymatic characteristics of cellobiose phosphorylase from *Ruminococcus albus* NE1 and kinetic mechanism of unusual substrate inhibition in reverse phosphorolysis. Biosci Biotechnol Biochem.

[18] Yernool DA, McCarthy JK, Eveleigh DE, Bok JD (2000). Cloning and characterization of the glucooligosaccharide catabolic pathway beta-glucan glucohydrolase and cellobiose phosphorylase in the marine hyperthermophile *Thermotoga neapolitana*. J Bacteriol.

[19] Wu Y, Mao G, Fan H, Song A, Zhang YP, Chen H (2017). Biochemical properties of GH94 cellodextrin phosphorylase THA_1941 from a thermophilic eubacterium *Thermosipho africanus* TCF52B with cellobiose phosphorylase activity. Sci Rep..

[20] Kitaoka M (2015). Diversity of phosphorylases in glycoside hydrolase families. Appl Microbiol Biotechnol.

[21] Sawano T, Saburi W, Hamura K, Matsui H, Mori H (2013). Characterization of *Ruminococcus albus* cellodextrin phosphorylase and identification of a key phenylalanine residue for acceptor specificity and affinity to the phosphate group. FEBS J.

[22] Hiraishi M, Igarashi K, Kimura S, Wada M, Kitaoka M, Samejima M (2009). Synthesis of highly ordered cellulose II in vitro using cellodextrin phosphorylase. Carbohydr Res.

[23] Bianchetti CM, Elsen NL, Fox BG, Phillips GN (2011). Structure of cellobiose phosphorylase from *Clostridium thermocellum* in complex with phosphate. Acta Crystallogr..

[24] O’Neill EC, Pergolizzi G, Stevenson CEM, Lawson DM, Nepogodiev SA, Field RA (2017). Cellodextrin phosphorylase from *Ruminiclostridium thermocellum*: X-ray crystal structure and substrate specificity analysis. Carbohydr Res.

[25] Heap JT, Pennington OJ, Cartman ST, Carter GP, Minton NP (2007). The ClosTron: a universal gene knock-out system for the genus *Clostridium*. J Microbiol Methods..

[26] Liu W, Bevan DR, Zhang YH (2010). The family 1 glycoside hydrolase from *Clostridium cellulolyticum* H10 is a cellodextrin glucohydrolase. Appl Biochem Biotechnol.

[27] Franche N, Tardif C, Ravachol J, Harchouni S, Ferdinand PH, Borne R (2016). Cel5I, a SLH-containing glycoside hydrolase: characterization and investigation on its role in *Ruminiclostridium cellulolyticum*. PLoS ONE.

[28] Ferdinand PH, Borne R, Trotter V, Pagès S, Tardif C, Fierobe HP (2013). Are cellulosome scaffolding protein CipC and CBM3-containing protein HycP, involved in adherence of *Clostridium cellulolyticum* to cellulose?. PLoS ONE.

[29] Petitdemange E, Caillet F, Giallo J, Gaudin C (1948). *Clostridium cellulolyticum* sp. nov., a cellulolytic mesophile species from decayed grass. Int J Sys Bacteriol..

[30] Giallo J, Gaudin C, Belaich JP, Petitdemange E, Caillet-Mangin F (1983). Metabolism of glucose and cellobiose by cellulolytic mesophilic *Clostridium* sp. strain H10. Appl Environ Microbiol..

[31] Celik H, Blouzard JC, Voigt B, Becher D, Trotter V, Fierobe HP, Tardif C, Pagès S, de Philip P (2013). A two-component system (XydS/R) controls the expression of genes encoding CBM6-containing proteins in response to straw in *Clostridium cellulolyticum*. PLoS ONE.

[32] Roy A, Kucukural A, Zhang Y (2010). I-TASSER: a unified platform for automated protein structure and function prediction. Nat Protoc.

[33] Zhang Y (2008). I-TASSER server for protein 3D structure prediction. BMC Bioinform.

[34] Emsley P, Lohkamp B, Scott W, Cowtan K (2010). Features and development of Coot. Acta Crist D..

[35] Zhang J, Liang Y, Zhang Y (2011). Atomic-level protein structure refinement using fragment-guided molecular dynamics conformation sampling. Structure..

[36] Notredame C, Higgins DG, Heringa J (2000). T-Coffee: a novel method for multiple sequence alignments. J Mol Biol.

[37] Robert X, Gouet P (2014). Deciphering key features in protein structures with the new ENDscript server. Nuclear Acids Res..

