# Supplementary material for: In vitro and in vivo exploration of the cellobiose and cellodextrin phosphorylases panel in Ruminiclostridium cellulolyticum: implication for cellulose catabolism
Source: Biotechnol Biofuels. 2019 Sep 3;12:208. doi: 10.1186/s13068-019-1549-x (PMC6720390; doi:10.1186/s13068-019-1549-x)
Supplement: Supplementary file 1 — Additional file 1. Purified recombinant phosphorylases. Samples of purified recombinant proteins (3 µg) were loaded on gradient 4–15% SDS-PAGE then Coomassie Blue stained. The recombinant cellobiose phosphorylase (CbpA) and CdpA, CdpB and CdpC have theoretical molecular weights of 93.5 kDa, 90.5, 91 and 94 kDa, respectively. [file 13068_2019_1549_MOESM1_ESM.pdf]

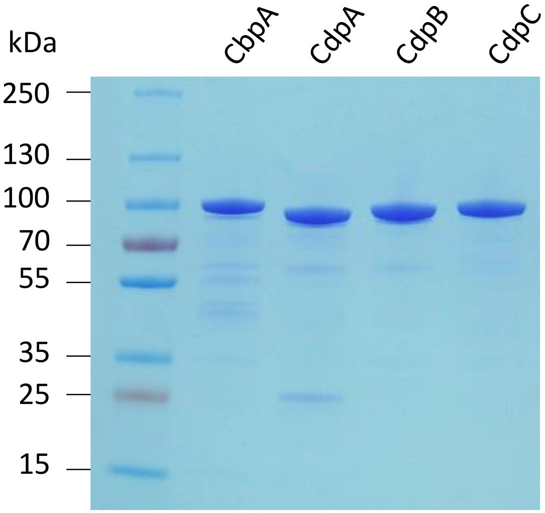


**Additional file 1. Purified recombinant phosphorylases.** Samples of purified recombinant proteins (3µg) were loaded on gradient 4-15% SDS-PAGE then Coomassie Blue stained. The recombinant cellobiose phosphorylase (CbpA) and CdpA, CdpB and CdpC have theoretical molecular weights of 93.5 kDa, 90.5, 91 and 94 kDa, respectively.
