# Supplementary figures and images for: In vitro and in vivo exploration of the cellobiose and cellodextrin phosphorylases panel in Ruminiclostridium cellulolyticum: implication for cellulose catabolism
Source: Biotechnol Biofuels. 2019 Sep 3;12:208. doi: 10.1186/s13068-019-1549-x (PMC6720390; doi:10.1186/s13068-019-1549-x)

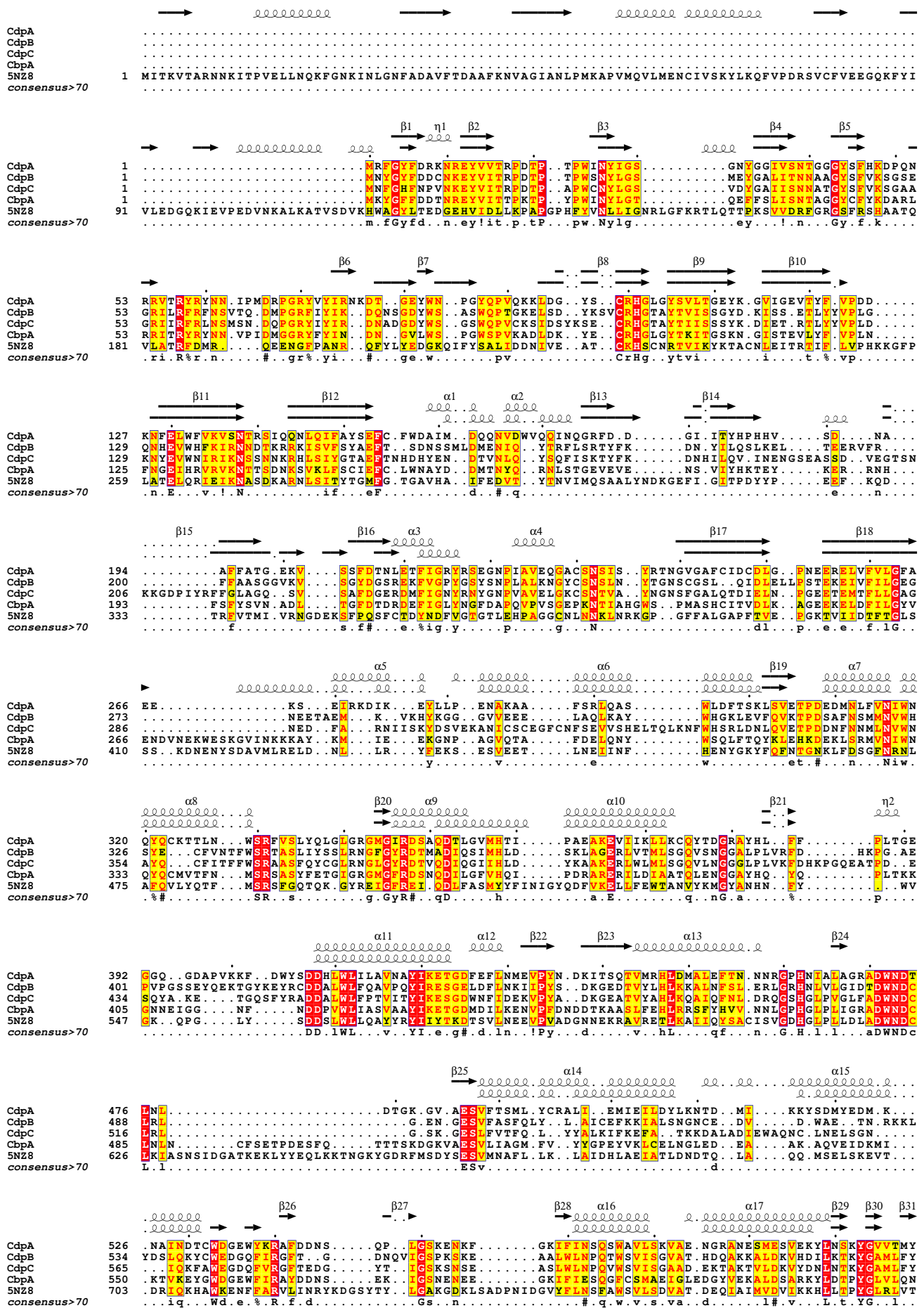

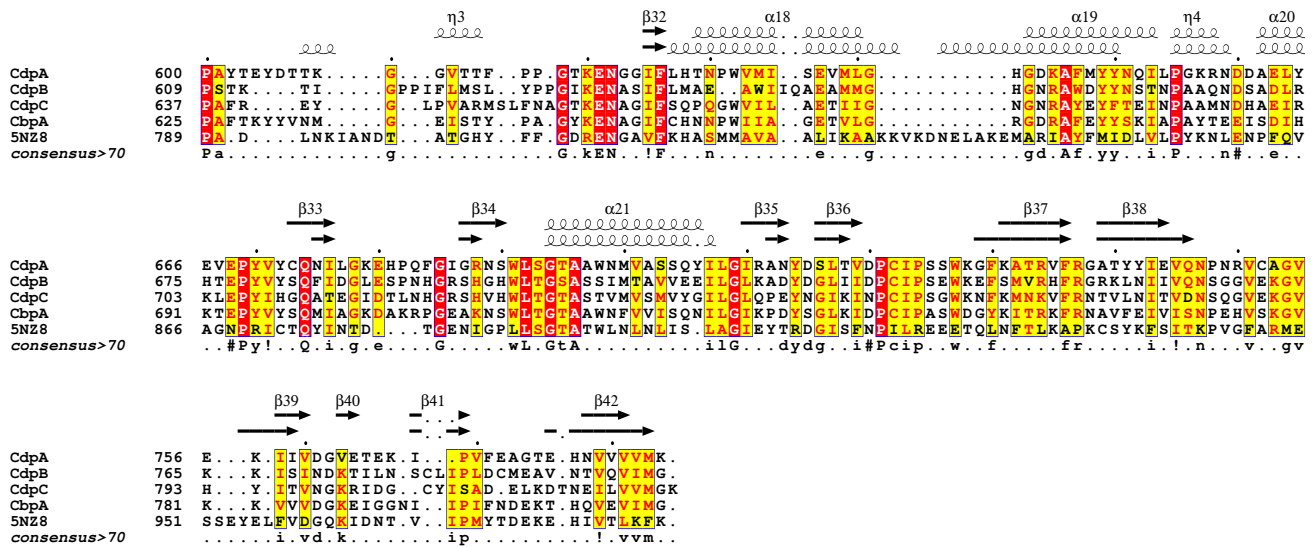

Supplement: Supplementary file 2 — Additional file 2. Sequence alignment of all modeled phosphorylases CdpA, CdpB, CdpC and CbpA from R. cellulolyticum and C. thermocellum cellodextrin phosphorylase (5NZ8). Secondary structure from 5NZ8 (lower line) and the CdpA model (upper line) are also indicated. [file 13068_2019_1549_MOESM2_ESM.pdf]
