# Supplementary material for: In vitro and in vivo exploration of the cellobiose and cellodextrin phosphorylases panel in Ruminiclostridium cellulolyticum: implication for cellulose catabolism
Source: Biotechnol Biofuels. 2019 Sep 3;12:208. doi: 10.1186/s13068-019-1549-x (PMC6720390; doi:10.1186/s13068-019-1549-x)
Supplement: Supplementary file 3 — Additional file 3. Molecular analysis of the Ruminiclostridium cellulolyticum mutant strains. A. Southern blot analysis of the strains. Genomic DNA or pMTLcbpA and pMTLcdpB were digested by PstI or EcoRI. After migration and transfert, the membrane was probed with a labeled probe targeting the erythromycine resistance cassette. The size of the detected fragments is consistent with theoretical sizes: MLTcbpA, 2.8 kb; MTLcdpA, 6.6 kb; MTLcdpB, 4.2 kb; MTLcdpC, 7.8 kb. B. PCR analysis of genomic DNA using primers hybridizing upstream and downstream the insertion site in the respectives target genes. Insertion of the intron increases the size by 1,78 kb in the mutant strains compared to the WT genomic DNA. [file 13068_2019_1549_MOESM3_ESM.pdf]

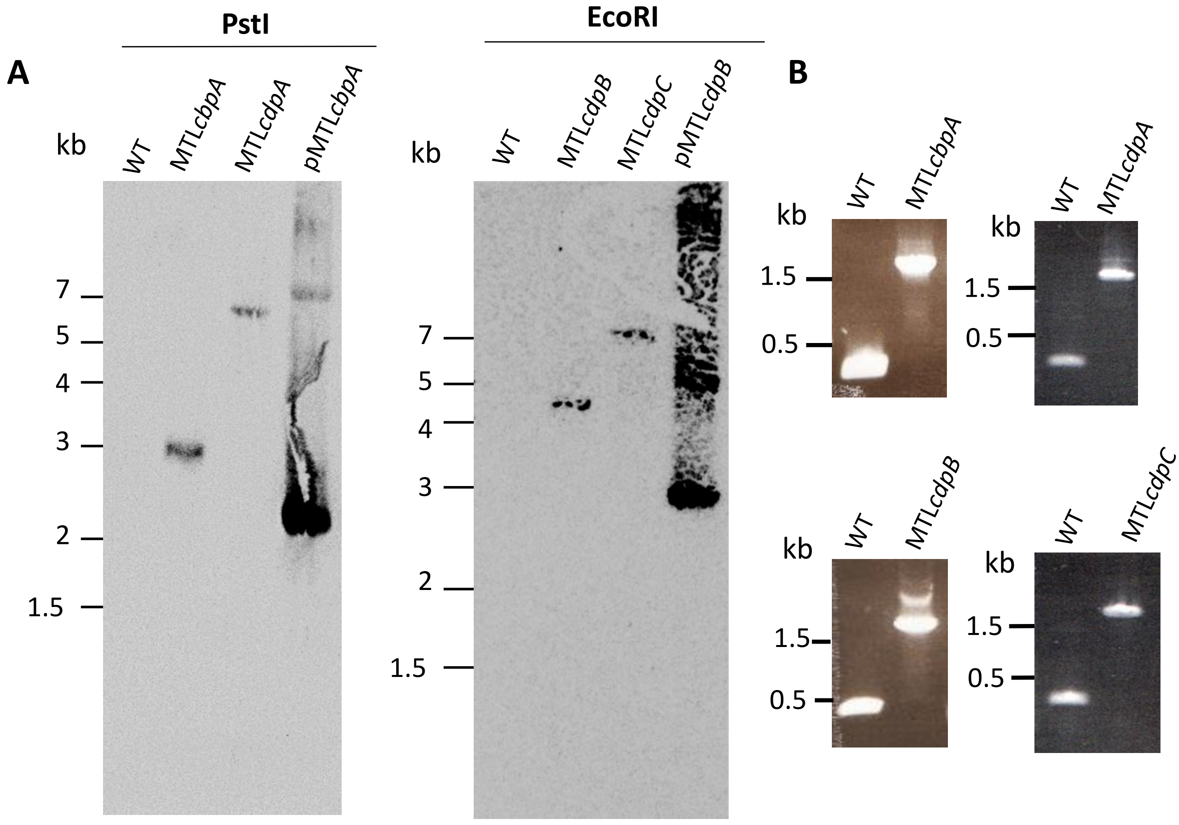

Additional file 3. **Molecular analysis of the *Ruminiclostridium cellulolyticum* mutant strains.**

A. Southern blot analysis of the strains. Genomic DNA or pMTL*cbpA* and pMTL*cdpB* were digested by PstI or EcoRI. After migration and transfert, the membrane was probed with a labeled probe targeting the erythromycine resistance cassette. The size of the detected fragments is consistent with theoretical sizes : MLT*cbpA*, 2.8 kb; MTL*cdpA*, 6.6 kb ; MTL*cdpB*, 4.2 kb ; MTL*cdpC*, 7.8 kb. **B.** PCR analysis of genomic DNA using primers hybridizing upstream and downstream the insertion site in the respectives target genes. Insertion of the intron increases the size by 1,78 kb in the mutant strains compared to the WT genomic DNA.
