# Supplementary material for: In vitro and in vivo exploration of the cellobiose and cellodextrin phosphorylases panel in Ruminiclostridium cellulolyticum: implication for cellulose catabolism
Source: Biotechnol Biofuels. 2019 Sep 3;12:208. doi: 10.1186/s13068-019-1549-x (PMC6720390; doi:10.1186/s13068-019-1549-x)
Supplement: Supplementary file 4 — Additional file 4. Growth of R. cellulolyticum wild-type, mutant and derivatives strains on arabinose The strains were grown on minimal medium containing 2 g L−1 arabinose. A. the strains are: WT (black) and mutant strains MTLcbpA (red), MTLcdpA (purple), MTLcdpB (blue) and MTLcdpC (green). B. The strains are: WT strain (black), WT strain carrying an empty vector (grey), MTLcbpA strain carrying an empty vector (pink), MTLcbpA strain carrying pSOScbpA (blue). Experiments were performed in triplicates and bars indicate standard deviation. [file 13068_2019_1549_MOESM4_ESM.pdf]

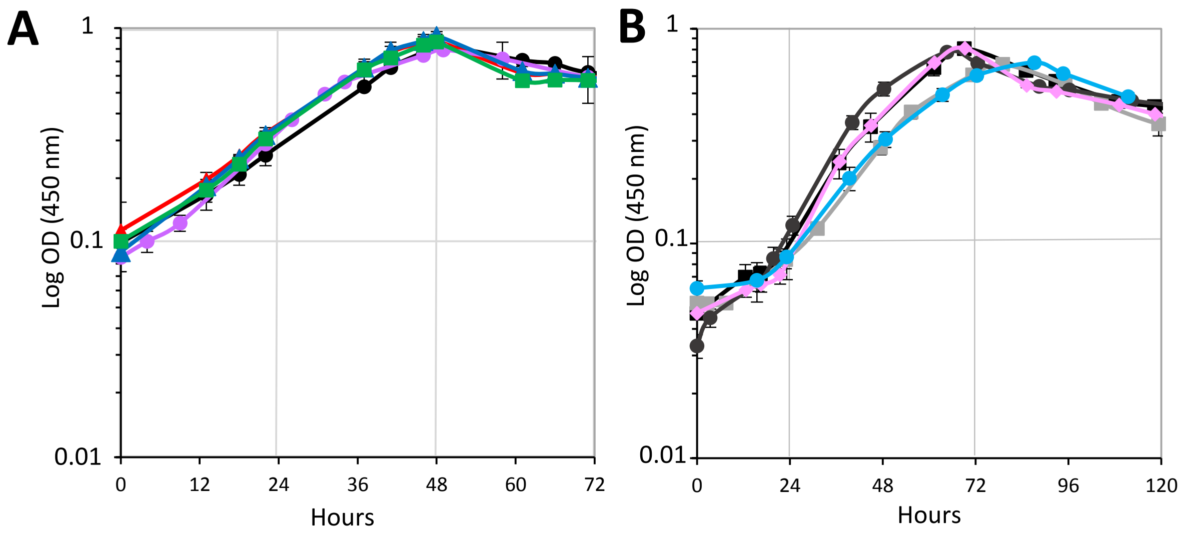

Additional file 4. Growth of *R. cellulolyticum* wild-type, mutant and derivatives strains on arabinose

The strains were grown on minimal medium containing 2 g L^{-1} arabinose. A. the strains are : WT (black) and mutant strains MTL*cbpA* (red), MTL*cdpA* (purple), MTL*cdpB* (blue) and MTL*cdpC* (green). B. The strains are : WT strain (black), WT strain carrying an empty vector (grey), MTL*cbpA* strain carrying an empty vector (pink), MTL*cbpA* strain carrying pSOS*cbpA* (blue). Experiments were performed in triplicates and bars indicate standard deviation.
