# Supplementary material for: In vitro and in vivo exploration of the cellobiose and cellodextrin phosphorylases panel in Ruminiclostridium cellulolyticum: implication for cellulose catabolism
Source: Biotechnol Biofuels. 2019 Sep 3;12:208. doi: 10.1186/s13068-019-1549-x (PMC6720390; doi:10.1186/s13068-019-1549-x)
Supplement: Supplementary file 5 — Additional file 5. Bacterial strains and vectors used. [file 13068_2019_1549_MOESM5_ESM.pdf]

Additional file 5. Bacterial strains and vectors used

Strain or plasmid	Relevant characteristics	Source or reference
E. coli DH5α	F^- *endA1 hsdR17*(rK^- mK^+) *supE44 thi-1* λ^- *gyrA96 relA1Δ*(*lacZYA argF*) *U169* (Φ80 *lacZΔ*M15) *recA*	Roche Diagnostics
E. coli BL21(DE3)	F^- *ompT hsdS* (rB^- mB^-) *gal dcm* (DE3)	Novagen
R. cellulolyticum	Wild-type, H10, ATCC35319, DSM 5812	DSMZ
R. cellulolyticum MTL*cbpA*	ATCC35319, *cbpA*::intron, Erm^r	This study
R. cellulolyticum MTL*cdpA*	ATCC35319, *cdpA*::intron, Erm^r	This study
R. cellulolyticum MTL*cdpB*	ATCC35319, *cdpB*::intron, Erm^r	This study
R. cellulolyticum MTL*cdpC*	ATCC35319, *cdpC*::intron, Erm^r	This study
pET22b(+)	*E. coli* expression vector, Amp^r	Novagen
pET*cbpA*	pET22b+ derivative carrying the NdeI-XhoI fragment encoding CbpA	[10]
pET*cdpA*	pET22b+ derivative carrying the NdeI-XhoI fragment encoding CdpA	This study
pET*cdpB*	pET22b+ derivative carrying the NdeI-XhoI fragment encoding CdpB	This study
pET*cdpC*	pET22b+ derivative carrying the NdeI-XhoI fragment encoding CdpC	This study
pMTL007	*E. coli/Clostridium* shuttle vector (ColE1, pCB102)Ll.*ltr*Bintron (*erm*BtdRAM2) under the control of P*fac*, *ltr*A; Cm^r/Tm^r	[25]
pMTL*cbpA*	pMTL007 derivative targeting *cbpA* (locus Ccel_2109)	This study
pMTL*cdpA*	pMTL007 derivative targeting *cdpA* (locus Ccel_1439)	This study
pMTL*cdpB*	pMTL007 derivative targeting *cdpB* (locus Ccel_2354)	This study
pMTL*cdpC*	pMTL007 derivative targeting *cdpC* (locus Ccel_3412)	This study
pSOSzeroTm	*E. coli/Clostridium* shuttle vector (ColE1, pIM13); Ap^r, Cm^r/Tm^r	[31]
pSOS*cbpA*	pSOS956 *E. coli/Clostridium* shuttle vector (ColE1, pIM13); Ap^r, Cm^r/Tm^r, derivative, carrying the BamHI-NarI fragment encoding full length *cbpA*, under the control of a weakened *Clostridium acetobutylicum thiolase* gene promotor (P_{thl})	[10]

Ap^r, ampicilline resistance; Erm^r, erythromycin resistance; Cm^r/Tm^r, chloramphenicol/thiamphenicol resistance
