# Supplementary material for: In vitro and in vivo exploration of the cellobiose and cellodextrin phosphorylases panel in Ruminiclostridium cellulolyticum: implication for cellulose catabolism
Source: Biotechnol Biofuels. 2019 Sep 3;12:208. doi: 10.1186/s13068-019-1549-x (PMC6720390; doi:10.1186/s13068-019-1549-x)
Supplement: Supplementary file 6 — Additional file 6. Primer sequences used in the present study. [file 13068_2019_1549_MOESM6_ESM.pdf]

Additional file 6. Primer sequences used in the present study

EXPERIMENT	NAME	SEQUENCE	FEATURES
**Targeted mutagenesis**	IBS*cbpA*_1668a	AAAAAAGCTTATAATTATCCTTATTCGCCGTCCCAGTGCGCCCAGATAGGGTG	
	EBS1d*cbpA*_1668a	CAGATTGTACAAATGTGGTGATAACAGATAAGTCGTCCCAACTAACTTACCTTTCTTTGT	
	EBS2*cbpA*_1668a	TGAACGCAAGTTTCTAATTTCGGTTGCGAATCGATAGAGGAAAGTGTCT	
	EBS universal	CGAAATTAGAAACTTGCGTTCAGTAAAC	
	IBS*cdpA*_537s	AAAAAAGCTTATAATTATCCTTAAGATTCGATGATGTGCGCCCAGATAGGGTG	
	EBS1d*cdpA*_537s	CAGATTGTACAAATGTGGTGATAACAGATAAGTCGATGATGGTAACTTACCTTTCTTTGT	
	EBS2*cdpA*_537s	TGAACGCAAGTTTCTAATTTCGATTAATCTTCGATAGAGGAAAGTGTCT	
	IBS*cdpB_53a*	AAAAAAGCTTATAATTATCCTTAGTAGGCGTATCGGTGCGCCCAGATAGGGTG	
	EBS1d*cdpB_53a*	CAGATTGTACAAATGTGGTGATAACAGATAAGTCGTATCGGGTAACTTACCTTTCTTTGT	
	EBS2*cdpB_53a*	TGAACGCAAGTTTCTAATTTCGATTCCTACTCGATAGAGGAAAGTGTCT	
	IBS*cdpC_480a*	AAAAAAGCTTATAATTATCCTTAATTTTCATAGTGGTGCGCCCAGATAGGGTG	
	EBS1d*cdpC_480a*	CAGATTGTACAAATGTGGTGATAACAGATAAGTCATAGTGGTTAACTTACCTTTCTTTGT	
	EBS2*cdpC_480a*	TGAACGCAAGTTTCTAATTTCGGTTAAAATCCGATAGAGGAAAGTGTCT	
**Control of the mutations**	2109_1510D	AAAGATGGGAAAGTGGCAGA	
	2109_1785R	CTCCGCCATAGAACAGAAGC	
	1439-454D	TCGGAGTTTTGCTTCTGGGA	
	1439-694R	TACTGCAAGCTCCCTGTTCT	
	2354_2D	TGAATTACGGTTATTTTGATGATTG	
	2354_261R	CCAGGAGGCTGACCAGTAAT	
	3412_298D	GAGTGCAGACATGGAACTGC	
	3412_583R	CGCTGCCGTTTTCATTTATT	
**Production in *E. coli***	1439NdeIdir	TTAACAT**ATG**AGATTCGGGTATTTTGACCG	NdeI is underlined, ATG boldface type
	1439XhoIrev	TTTTCTCGAGTTTCATTACAACGACTACATTGTGC	XhoI is underlined
	1439_a951t_rev	ATACTGATTCCAAATGTTTACAAACAGATTC	
	1439_a951t_dir	TTTGTAAACATTTGGAATCAGTATCAGTGC	
	2354NdeIdir	TTAACAT**ATG**AATTACGGTTATTTTGATGATTGT	NdeI is underlined, ATG boldface type
	2354XhoIrev	TTAACTCGAGACCCATAATTACCTGAACAGTATTTAC	XhoI is underlined
	3412NdeIdir	TTAACAT**ATG**AATTTTGGTCATTTTAATCCAGT	NdeI is underlined, ATG boldface type
	3412XhoIrev	ATTTCTCGAGCTTACCCATTACTACTAATATTTCATTAGTATC	XhoI is underlined

EXPERIMENT	NAME	SEQUENCE	FEATURES
**qRT-PCR**	16SFrw	CTATGTTTCTTGAGTGCCGG	
	16Srev	ATACTTATTGTTGTAACTCCGG	
	2109F	ATGAGTGCAGGCATGGTTTG	
	2109R	ACCGAGTTCTCAACTTCAACC	
	q1439_454D	TCGGAGTTTTGCTTCTGGGA	
	q1439_694R	TACTGCAAGCTCCCTGTTCT	
	q2354_374D	TTCCGTTGGGTCAAAACCAC	
	q2354_589R	CCCTTTCCTCGGTCAGTTCT	
	q3412_456D	TACGGCGGAATTCACAAACC	
	q3412_676R	CGAAAGCAGAAACGGACTGG	
