# Supplementary material for: In vitro and in vivo exploration of the cellobiose and cellodextrin phosphorylases panel in Ruminiclostridium cellulolyticum: implication for cellulose catabolism
Source: Biotechnol Biofuels. 2019 Sep 3;12:208. doi: 10.1186/s13068-019-1549-x (PMC6720390; doi:10.1186/s13068-019-1549-x)
Supplement: Supplementary file 7 — Additional file 7. Experimental conditions used for enzymatic parameter measurement. The tables show the initial velocities measured for each cellodextrin phosphorylase and substrate using experimental conditions obtained after optimization of the enzyme concentrations and the time points. [file 13068_2019_1549_MOESM7_ESM.pdf]

Additional file 7. Experimental conditions used for enzymatic parameter measurement.

The tables show the initial velocities measured using optimized experimental conditions (enzyme concentrations and time points) for each cellodextrin phosphorylase and substrate.

Enzyme concentration	**CdpA** 0.1 µM						
Substrate	Cellotetraose						
Substrate concentration (mM)	20	10	5	3.33	1	0.4	0.2
Incubation time (minutes)	10						
Cellotriose increase (µM/min)	112.2±3.3	181.9±6.2	103.3±2.2	49.6±2.6	17.5±0.2	7.2±0.4	3.9±0.1

Enzyme concentration	**CdpA** 0.1 µM					
Substrate	Cellopentaose					
Substrate concentration (mM)	8.48	5	3.33	1	0.4	0.2
Incubation time (minutes)	5					
Cellotetraose and cellotriose increase (µM/min)	176.2±18.5	134.1±11.8	126.2±4.3	39.9±1.8	19.5±0.2	9.9±0.7

Enzyme concentration	**CdpB** 2 µM				
Substrate	Cellotetraose				
Substrate concentration (mM)	10	5	3.33	0.2	0.133
Incubation time (minutes)	10				
Cellotriose increase (µM/min)	100.1±5.2	73.0±3.5	57.1±13.4	4.5±0.2	3.5±0.3

Enzyme concentration	**CdpC** 0.1 µM						
Substrate	Cellotriose						
Substrate concentration (mM)	20	10	5	3.33	1	0.4	0.2
Incubation time (minutes)	5						
Cellobiose increase (µM/min)	780.9±10.0	536.8±47.1	389.6±5.8	293.3±3.0	114.2±10.1	50.3±0.8	27.6±0.5
